# Enablers and Barriers to an Experience‐Based Co‐Design Process to Develop Service Improvements in Enhanced Community Care in Ireland: A Qualitative Study

**DOI:** 10.1111/hex.70206

**Published:** 2025-03-12

**Authors:** Fay O'Donoghue, Máire T O'Donnell, Tomás P. Griffin, Eileen Fahy, Elaine Newell, Ann‐Marie Creaven

**Affiliations:** ^1^ Department of Psychology University of Limerick Limerick Ireland; ^2^ School of Medicine University of Galway Galway Ireland; ^3^ Centre for Diabetes and Endocrinology Galway University Hospital Galway Ireland; ^4^ Community Healthcare West, HSE Galway Ireland; ^5^ Health Research Institute University of Limerick Limerick Ireland

**Keywords:** Enhanced Community Care, experience‐based co‐design, healthcare, integrated care, patient experience, qualitative, service user

## Abstract

**Background:**

Experience‐Based Co‐Design (EBCD) is a popular collaborative process where service users and healthcare providers share their experiences of using and delivering services to identify ways to adapt services to enhance those experiences.

**Objective:**

This study aimed to identify enablers and barriers to the successful implementation of EBCD as part of Ireland's recently adopted Enhanced Community Care (ECC) programme.

**Design:**

Service users and staff at two sites (*N* = 17) participated in an accelerated EBCD process designed to enhance service provision for older people and those living with chronic conditions. This included four co‐design working group sessions per site.

**Methods:**

Transcripts from the co‐design working groups and from brief follow‐up interviews with individual participants were analysed. Thematic analysis was used to identify enablers and barriers to the EBCD process.

**Results:**

We generated six key themes reflecting barriers and enablers; enablers were *the fundamental role of the facilitator* (Theme 1), *a flexible approach that met group members' needs* (Theme 2) and *active and interactive activities to support participant engagement* (Theme 3). The *fundamental role of the facilitator* was also identified as a barrier (Theme 4); additional barriers included *balancing experience‐sharing and decompressing* (Theme 5) and *the scope of the group as an invisible barrier* (Theme 6), which reflected challenges in facilitating dialogue about change when participants were aware of system‐level constraints on the potential for change.

**Conclusions:**

The facilitator is critical in ensuring the successful implementation of the EBCD process. Considering how best to draw on the facilitator strengths while also ensuring that the service user perspectives are equally weighted with staff perspectives is important for effective communication within EBCD projects.

**Patient or Public Contribution:**

Service users (also including carers) at two sites participated in EBCD projects alongside health and social care professionals, ultimately generating two service improvements for the ECC programme. The participation of these service users was celebrated at an academic conference, which was attended by a number of service users, and where the outcomes of the EBCD project were presented.

## 1 Introduction

Experience‐based co‐design (EBCD) is a quality improvement process that brings together service users and healthcare professionals to understand how service users experience services and how staff experience the delivery of that service (e.g., Boyd et al. [1]). This shared understanding is intended to improve health and social care experiences, systems and processes. In an EBCD project, service users share their health and social care experiences, identify priorities for the improvement of these experiences, develop potential solutions in conjunction with staff, and at times contribute to the implementation and evaluation of these solutions. The popularity of EBCD reflects increasing recognition that public and patient involvement in healthcare processes can be valuable in enhancing the quality of services offered. Since Boyd et al. [1] described their six‐step process of implementing EBCD (these six steps being, to engage, plan, explore, develop, decide and change), variations in EBCD approaches have been used to enhance service provision across multiple areas, including for families of children with type 1 diabetes [2], heart failure care [3] and heart failure medication management (e.g., Raynor et al. [4]). However, most studies in one recent review [5] were conducted in acute hospital settings in the United Kingdom, with only one reported study in primary care for service users with multiple morbidities [6]. Therefore, relatively less is known about how EBCD approaches might be applied in integrated care settings. Importantly, in 2020, the Irish Health Service Executive (HSE) launched the Enhanced Community Care (ECC) Programme, which aimed to deliver a multi‐disciplinary, integrated approach to care for older persons and people living with chronic conditions by bridging the gap between primary and secondary care (HSE, [7]). The new community‐based specialist teams operate in Ambulatory Care Hubs and work closely with General Practitioners and Practice Nurses with the aim of shifting care away from hospitals by meeting the needs of these populations in primary and community care settings where possible. This programme was developed in response to the anticipated healthcare needs of an ageing population and the increasing prevalence of chronic conditions, as well as in response to service users' preferences. Owing to Covid‐19, ECC implementation was accelerated, and the programme has transformed how healthcare is delivered in Ireland in a relatively short space of time. There is a strong emphasis on the prevention of chronic conditions, early intervention and self‐care support. An ECC programme was introduced in our local area (the West of Ireland) in 2021, and the EBCD approach was adopted to explore how services could be enhanced in this new ECC context. Our current study complements the literature on EBCD studies in hospital settings, by exploring enablers and barriers to the EBCD approach in the ECC context. While some studies have detailed the outcomes of applying EBCD to identify service improvements, fewer studies explore the enablers and barriers to its effective implementation. In the broader EBCD context, Fylan et al. [8] highlighted the critical role of trigger films (a film of care experiences to stimulate discussion and generate perspectives) in fostering a common purpose between service users and staff, ensuring a focus on the overall objective of improved care. Moser et al. [9] revealed challenges, including the reflective capacity of older service users, securing professional commitment, overcoming cultural differences and involving researchers and facilitators with appropriate expertise. Ramos et al. [10] interviewed seven EBCD facilitators, identifying the facilitator's role, organisational support, narrative‐based interaction, pre‐intervention preparation, service type and existing relationships with service users as enablers. They also identified barriers, including the impact of service users' conditions or carers' schedules on participation, staff/management resistance, workload concerns, time constraints, organisational barriers such as the healthcare system's limited capacity to adapt and power dynamics. Power imbalances may arise between researchers and participants or among participants themselves, influenced by factors such as roles, class, skills, language and participatory environments that may or may not make individuals feel comfortable contributing [11]. As Roura [12] highlights, power in participatory health research is shaped by individual, interpersonal and structural factors. For example, patients may hesitate to critique healthcare systems in the presence of clinicians due to perceived authority gaps, while clinicians might inadvertently dominate discussions, prioritising professional concerns over patient needs. The location, structure and facilitation of participatory sessions can also impact power dynamics, either empowering or marginalising voices. Clearly, there are significant enablers and barriers affecting EBCD success at both individual and organisational levels; however, relatively little has been published relating to EBCD in integrated care. Our analysis is the first examination of the enablers and barriers to implementing the EBCD approach to identify service improvements in the ECC context.

## 2 Methods

### 2.1 Description of EBCD Project

Here we outline the overall EBCD project methodology before outlining the evaluation methodology.

#### 2.1.1 The EBCD Project

**Figure 1:**
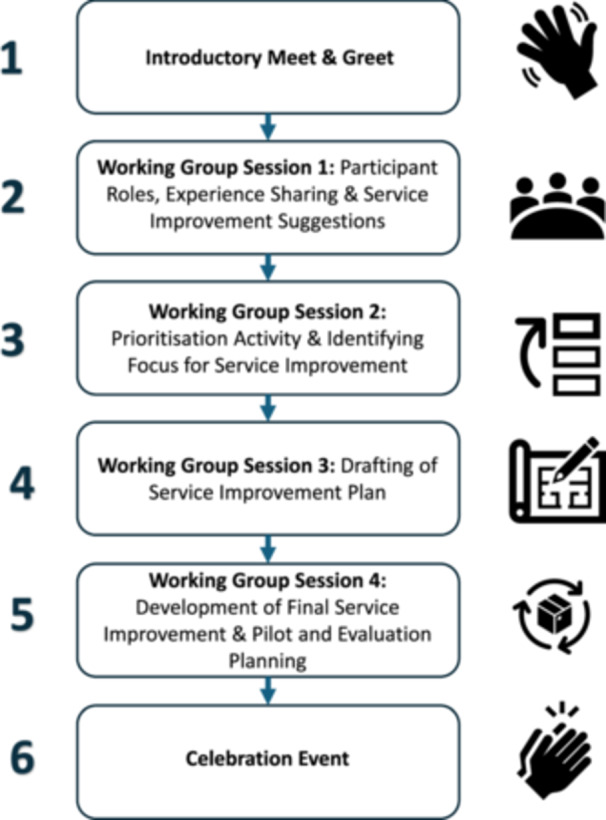
Visual overview of the accelerated EBCD process adopted.

The aim of the ‘Better Together’—Transforming Community Care project was to use the EBCD process to aid service users, healthcare providers and other staff in working together in co‐designing and implementing a community service improvement important to these groups, across two separate ECC sites. Service users included people living with chronic conditions (type 2 diabetes, congestive cardiac failure and chronic obstructive pulmonary disease) and older persons (> 75 years of age) supported through community care. This project adopted an accelerated EBCD process [11] (see Figure 1) and was informed by the design thinking literature [13]. This outlines an iterative process of empathising (observing and engaging with users), defining (creating an actional problem statement that reflects group needs), ideating (generating ideas), prototype development (generating artefacts [e.g., notes or visual aids] that move closer to a solution) and testing (soliciting feedback), to achieve a design goal. All sessions involved joint discussions with staff, service users and carers and were facilitated by one of two facilitators, both with healthcare expertise; one was the project lead (E.N.) and the other was recruited through the lead's professional networks. External facilitation was considered; however, partly owing to resource constraints, this was not possible; additionally, co‐facilitation with service users was not feasible as the requisite facilitation experience was not evident in the participating cohorts. Commencing January 2023, sessions for both groups occurred at a neutral hotel venue, lasting approximately 2 h each. Ethical approval was obtained from the Galway Clinical Research Ethics Committee (CA 2902).

***Meet and greet:*** Before the meet‐and‐greet, service users and carers completed a readiness assessment and received project details. The assessment prompted reflection on their interest and capacity to engage (e.g., endorsement of statements like ‘I have time for user engagement’; ‘I respect others' perspectives’). During this event, short trigger videos showcased local care experience stories. Participants reported that these videos were effective in sparking impactful discussions regarding care experiences and in encouraging diverse perspectives. Attendees expressing interest provided written consent and scheduled subsequent sessions. A ‘Sharing Your Story—Key Points about Your Care Experience’ document (Supplementary Material 1), developed specifically for this project, was shared with service users before the first session. This supported service users to reflect on and prepare their account of their care experience in advance, to facilitate concise and focused accounts during the first session.

1. ***Co‐design working group 1:*** This session began by outlining the project parameters and participants' roles. Participants voiced what was important to them in developing and managing group needs (i.e., encourage equal participation; respect confidentiality; maintain a respectful, punctual and non‐judgmental environment). Following this, all participants were asked to share an example of when a care experience/experience of delivering care went well, and an example of when an experience did not go well, referring to their ‘Sharing Your Story’ document if needed. These experiences were openly discussed amongst the group. Finally, participants were asked to share any service improvement ideas that they had based on their own experiences and what had been discussed in the groups. All the touchpoints and key issues raised, as well as service improvement ideas shared, were tracked on a flip chart. Following Session 1, participants received written summaries and were encouraged to prioritise issues for the next session. Following Sessions 1, 2 and 3, all participants received brief follow‐up phone calls to provide ongoing evaluation of their participation (e.g., How was your experience of the session? Is there anything you would have done differently or would like done differently for the next session?).
2. ***Co‐design working group 2:*** Session 2 provided an overview of the first session's points, addressing key issues raised and collaboratively discussing how these could be addressed. Prioritisation tools from the literature were considered; however, as these were somewhat taxing for the service users within the current study, a bespoke prioritisation tool was used to map touchpoints, key issues and service improvement ideas. These were compiled into a list of concerns and potential improvements, which group members reviewed before the next session. Each member identified their top three priorities, assigning rankings of 1, 2 and 3, which were visually represented, on flip charts, during the session. The group collaboratively brainstormed feasible changes within their scope and at the service level, refining the list into a concise set of priorities. Ultimately, the group organically identified a single service improvement that addressed all their key priorities. The session concluded with the group choosing a service improvement idea (assessment discharge report template and resources for communication and person‐centred care). Following the session, participants received a written summary and were asked to gather resources for the agreed‐upon improvements.
3. ***Co‐design working group 3:*** The third co‐design working group began by giving an overview of the service improvement idea discussed in Session 2. The group shared the resources they had pooled between sessions (e.g., written templates, leaflets and brochures, obtained by both service users and staff). The group collaborated to pinpoint useful aspects of the gathered resources for their service improvement idea, resulting in a rough draft of the service improvement by the session's end. The facilitator documented and shared this draft with participants afterwards.
4. ***Co‐design working group 4:*** The last co‐design session began with participants sharing initial thoughts on the draft service improvement. Collaboratively, the group made changes based on shared opinions. After developing a final draft, they discussed pilot and implementation plans. The final draft was shared, and the session concluded with participants reflecting on the overall EBCD process.
5. ***Celebration event:*** The final stage was a celebration of the project outcomes. To achieve this, the project was presented at the first Patient Partnership Conference in October 2023; this included the project lead, an additional staff member and four service users.

#### 2.1.2 Participants

Participants were recruited using purposive sampling (see Table 1 for participant characteristics). Service user participants in Group 1 were recruited from a previous public service user engagement event that they were involved in, which included older people; people living with COPD, asthma, diabetes or a heart condition; and carers for people with one or more of these conditions. Service user participants in Group 2 were approached through an ECC programme for older persons. Group 1 included four service users who live with chronic conditions and four staff working in the ECC programme. Healthcare staff were recruited from a previous staff engagement event; their participation was entirely voluntary. Group 2 included two service users, three carers and four staff who work in the Integrated Care Programme for Older Persons (ICPOP) service, with different health and social care backgrounds.

**Table 1:** Participant characteristics for each EBCD working group.

Group 1 (Site G)			Group 2 (Site B)		
ID	Sex	Participant	ID	Sex	Participant
P1	Female	Service user	P1	Male	Carer
P2	Female	Service user	P2	Female	Staff
P3	Female	Staff	P3	Female	Staff
P4	Female	Staff	P4	Male	Service user
P5	Female	Staff	P5	Male	Carer
P6	Female	Service user	P6	Female	Staff
P7	Female	Staff	P7	Female	Carer
P8	Female	Service user	P8	Female	Staff
			P9	Female	Service user

*Note:* Service users were aged 65 years and over; to reduce participant burden and to reduce the risk of participation identification, no further demographic information was collected from service users.

#### 2.1.3 EBCD Project Outcomes

As a result of the project, Group 1 developed the ‘Better Together Transforming Community Care’ poster, leaflet and feedback form, with a specific pilot site identified for initial implementation and subsequent evaluation. The poster and leaflet with the feedback form allude to better communication and person‐centred care aimed at both staff and service users. Group 2 introduced the Community Specialist Team for Older Persons Assessment Report; this was an assessment report template designed for service users to retain and refer to, which contained key information, including who referred the individual to the service and the reason for referral, and a summary of the service users own care priorities. The project aimed to implement this service improvement across other ICPOP teams.

### 2.2 Description of Project Evaluation

#### 2.2.1 Data Collection

The co‐design working groups were recorded and transcribed; the additional individual phone interviews (6–8 per session per site) were conducted by an independent researcher (E.F.) who was not involved in the EBCD process and who was provided with a topic guide. Collectively, these transcripts formed the data for analysis of the EBCD process.

#### 2.2.2 Data Analysis for Qualitative Evaluation

The goal of this analysis was to identify enablers and barriers to the implementation of the EBCD approach in ECC. The analysis was led by the first author (F.O'.D.) in collaboration with A.M.C.; neither was involved in group facilitation. First, F.O'.D. and A.M.C. reviewed samples of the group and individual transcripts to consider the most appropriate analytical approach; we determined that the data were sufficiently ‘thick’ as to facilitate thematic analysis. Thematic analysis provides a systematic and flexible framework to analyse qualitative data by identifying and interpreting recurring patterns or themes within the transcripts and thus develop meaningful insights and a comprehensive understanding of the data. An inductive and data‐centred strategy was used. Once this strategy had been established, F.O'.D. immersed themselves in the transcripts through multiple iterations of listening, reading and re‐reading while taking preliminary notes. This process facilitated becoming acquainted with the data and transitioned the evaluator (F.O'.D.) into the analytical phase. Subsequently, employing a line‐by‐line latent coding approach, initial themes were derived. These themes represented data‐driven patterns which offered meaningful interpretations of the data in relation to the evaluation research questions [14]. Subsequently, these themes underwent an iterative process of development, review, refinement and definition, led by F.O'.D; A.M.C. discussed coding and theme development and interrogated for alternative interpretations. Occasionally, we spoke with a working group facilitator who offered useful context to the transcripts; for example, we noted that a particular participant had little to contribute during one working group session, and the facilitator noted that this participant felt unwell on that date and took a break from the working group. These conversations were useful in providing additional context to the analysis. Each theme was then assigned a descriptive name, encapsulating its core concepts. The transcripts were coded in their entirety in that service user and staff transcripts were not analysed separately; however, we included examples of quotations from both groups in our reporting.

## 3 Results

### 3.1 Enablers for and Barriers to the EBCD Process

**Figure 2:**
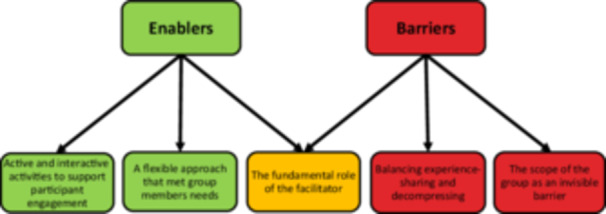
Visual overview of the identified enablers and barriers to implementing EBCD in ECC.

Three main themes were generated from the data, representing EBCD *enablers*, which contributed to participants' engagement and enjoyment of the EBCD process and aided the development of tailored service improvements. Additionally, we generated three themes representing barriers to EBCD implementation (see Figure 2). These themes were the fundamental role of the facilitator; this was identified as both an enabler and a potential barrier in our analysis. Additional enablers were a flexible approach that met group members' needs and active and interactive activities to support participant engagement. Additional barriers were balancing experience‐sharing and decompressing, and the scope of the group as an invisible barrier.

#### 3.1.1 Enablers

1. **The fundamental role of the facilitator**
  This theme represents the key role the facilitator has in the EBCD process. Participants described several examples of the facilitator positively impacting their involvement and experience of the EBCD process. For example, active listening and feeding back information shared to individuals ensured that the facilitator had accurately captured what the participants had shared and resulted in individuals feeling heard and valued:
*Facilitator: ‘Service User’, that's really valuable what you've shared there. I think what I can hear from you is that when you feel like you're treated like a person and you have time, it makes all the difference in the world and helps your experience of care.*
  Group 1; Session 1
  Participants commented on how the facilitator positively impacted their involvement, both in the working group and interview transcripts.
*Facilitator: So I'm hearing two things from that. Firstly, uh, how you feel and how you feel you're treated is a very important aspect of care within it*.
*Service User (P1): Well, a lot is down to you ‘Facilitator’. I mean, you've been patient, you've been a very good facilitator. Excellent because I've been to a lot of stuff now and I can be very critical, you know, and I do admire you. Yeah, I do. Yeah.*
  Group 1; Session 4
  This was also echoed by participants in the individual interviews:
*Interviewer: Did you feel listened to?*
*Staff (P5): Yeah, Definitely yeah yeah, I got lots of times to speak and ‘Facilitator’ sort of reiterated what I said, they asked ‘I am hearing’. They sort of repeated what I said and made sure that what I was trying to say came across, yeah definitely they were a very good host. Yeah.*
  Staff interview (Group 2; after Session 1)
*Interviewer:…and that brings me again very nicely to did you feel your contributions were heard and valued?*
*Service User (P6): Yes, and because everybody has different things to say and I certainly felt what I was saying was heard. And I had feedback from ‘Facilitator’ to ask me further questions about what I have said. And so just from that feedback, you know, they've heard what it is that you're you're speaking.*
  Service user interview (Group 1; after Session 1)
  Additional facilitator skills included, specifically going around the table and directly asking questions and individuals for input to ensure everyone is given the opportunity to share:
*Staff (P3): We were going around in a circle, kind of next person, next person, but if someone then hadn't spoken, the facilitator kind of saw that and just said, you know, called for the persons' name and said would you like to add anything, that kind of thing. So that was, that was really helpful.*
  Staff interview (Group 1; after Session 1)
*Carer (P1): The facilitator was reflecting feelings but doing it in a very accurate way, so when you said something, when they reflected it back to you, it was really what you meant them to understand.*
  Carer interview (Group 2; after Session 1)
  As well as this the facilitator steered the group to ensure that the group remained focused broadly on the EBCD goals. This steering also mitigated against staff potentially dominating conversations and highlighted to service users and carers that their opinions and ideas were valued:
*Facilitator: I am conscious we have a lot of staff talking and we just need to balance it and be mindful of our service users here as well.*
  (Group 2; Session 3)
  Finally, the facilitator identified when the group had reached a point where the work was becoming overly challenging and an agreement was not being reached and, thus, brought such conversations to a close. This may have reduced participant burn‐out and loss of engagement:
*Facilitator: While we are standing, it might be a good time to just have a look at the board and see how we're going so far and just do a quick a check in. We can have a quick look at what we've discussed so far and see are we on the right track.*
  (Group 2; Session 3)
  During one of the later co‐design workshop sessions in Group 1, the participants engaged in a prolonged discussion about the title of a theme for their poster, which was being developed as part of their service improvement project. Sensing that the discussion was becoming drawn out, the facilitator intervened and suggested temporarily choosing one of the identified theme names to facilitate progress. This decision enabled the group to shift their focus to developing content for the selected theme, preventing them from getting stuck at this initial stage and ensuring that the co‐design working group continued smoothly and finished on time as had been agreed at the outset of the project.
*Facilitator: We'll go with that theme for now. Let's just do a few bullet points on that one and then we'll finish up.*
  (Group 1; Session 3)
  Collectively, participants' discussions as part of the EBCD process, and the subsequent follow‐up interviews, indicate that the facilitator plays a major role in effectively implementing the EBCD process and optimising the outcomes from the group sessions.
2. **A flexible approach that met group members' needs**
  This theme represents the value in allowing participants to engage with the EBCD process in a way that suits them and outside of the group sessions when possible and convenient for participants:
*Facilitator: If you're a person that likes to be looking at things in between meetings, you have a look at them. If you'd rather do the work when we come here, I'm fine with that too. OK, whatever works for you guys because you know you're giving me your time.*
  (Group 2; Session 1)
  This flexibility resulted in participants actively engaging with the process. This was particularly evident when participants gathered resources for the co‐design working group in between sessions and thought about the co‐design working groups at certain points within their own lives, outside of the sessions. For example, one staff participant photographed a service improvement they encountered while attending an appointment with a family member. Service users also brought ideas from their ongoing care:
*Service User (P4):… I was at the hospital the other day at the new outpatient's department. I like the idea of the way they do it because you can't book into the hospital now for half an hour before your appointment, and when your appointment comes, you are called within a few minutes of your appointment time. I like that idea, because everybody's not rushing to the outpatients at the same time…So I think from that point of view, the idea of the new computer ID and getting the number is good.*
  (Group 2; Session 4)
  Additionally, the continued sharing of the work completed following each co‐design working group facilitated engagement outside of the group sessions and resulted in participants having ideas around draft improvements ready to go within the next session.
*Facilitator: Ok, so I sent you a poster and I sent you a leaflet, and it was just an initial draft. I was trying to put a bit of shape on it from all the valuable stuff you were giving me. As I said to you before, we will be getting help with the design of it, you know. So I was working off the printer at work so the colours are not great, but you need a visual so you can see something laid out. What was your initial impression of the poster?*
*Service User (P2): Oh it was amazing*.
*Staff (P5): It looks good. You know when it is made bigger, will there be a lot of space on it and do we need to just fill it up?*
*Staff (P4): I'd be worried about having too much on it as well*…
  (Group 1; Session 4)
3. **Active and interactive activities to support participant engagement**
  The final enabler captures something that was often mentioned by participants within the individual interviews, which was the use of certain interactive activities, particularly the prioritisation activity. This involved ranking the issues raised based on which issues individuals felt were most important to address. This prioritisation activity, as well as the constant visual representation (on flip charts) of the work completed, aided participants in visualising what was most important to the group and ensured this was a key focus throughout the co‐design working groups, optimising the EBCD process.
*Staff (P7): The facilitator put paper up along the walls so it was very visual. It was right there in front of us with all of the points. And then what I found really useful, we were all given sticky notes and we identified the three things that we felt were most important and we numbered them 1,2 and 3. And then it was a very good visual representation. You could see how many people felt that this particular point was the most important and so on. And I just found that really really helpful.*
  Staff interview (Group 1; after Session 2)
  Interviewer: Good. And what did you find useful in the session?
*Service User (P4): Well, I found everything useful but the chance of putting things up on the the White board is, yeah, you know, to put them up and get our views across. So I think it's quite handy and good you know.*
  Service user interview (Group 2; after Session 2)
*Staff (P7): I would be kind of a visual person anyway, and I think everybody, from what I can gather, benefited from that all being up around the room.*
  Staff interview (Group 1; after Session 2)
  Other activities that promoted a collaborative working environment and the development of the service improvements included movement breaks and the sharing of different resources which would be useful to each service improvement idea and then pulling pieces from these resources as a group. In brief, the use of interactive and active activities resulted in a collaborative working environment and increased the strengths of using the EBCD process.

#### 3.1.2 Barriers

Our analysis identified three barriers: these were (1) the fundamental role of the facilitator, (2) balancing experience‐sharing and decompressing and (3) the scope of the group as an invisible barrier.

1. **The fundamental role of the facilitator**
  Although identified as an enabler, the facilitator role could also potentially be a barrier to the efficacy of the EBCD process on occasion, where the facilitator almost steps outside the facilitator role to become an active participant within the group, particularly in terms of relating to staff. For example, one transcript contained a protracted staff discussion about the structure of a referral form that ideally would have occurred outside of the working group. Additionally, on occasion, the facilitator took a relatively active role in the development of ideas or decisions regarding the final product. For example, in the below extract, the facilitator notes that they *don't want to influence in regard to medication*, demonstrating awareness of the importance of their facilitator role, but also notes from their professional expertise that medication is an important topic for the discharge report form being generated.
*Facilitator:…the discharge letter is a snapshot in time and I don't want to influence in regards to medication, but I often feel that your community pharmacists and local pharmacist who deals with it, it's probably the key person in regards to medication because no matter where you are as a service user, whether you were in the hospital with our team or with the GP, the pharmacist is the first to receive it if there are any changes to medications. I do think the pharmacy is probably the key discipline for that. So for this letter, do we allude to that? You know, because we are supposed to do this in practice, make sure that they understand their medications. Do we just have a tick box to say refer to your medication list and that you go to your pharmacist or your doctor, and you get a handle on your medications and have the list recorded.*
  (Group 2; Session 3)
  Although this form of involvement is a potential barrier, some degree of expertise is needed to ensure the EBCD process progresses in a timely manner, and participants did appreciate the sense that a facilitator was on this journey with them:
*Service User (P6): She's [facilitator] like part of the group as well as opposed to somebody who's above us. Great. Really. Yeah.*
  Service user interview (Group 1, after Session 2)
  This demonstrates the challenge of the facilitator role in balancing the creation a comfortable environment where no one is ‘above’ the group while also avoiding becoming an active participant within the group.
2. **Balancing experience sharing and decompressing**
  This theme captures the challenge in balancing experience‐sharing and extended emotional expression in EBCD working groups to ensure that participants feel heard and that the project goals are met within the limited timeframe. As noted, the facilitator used a ‘Sharing Your Story’ tool to support participants in articulating their care experiences to the wider group. Although this scaffolded participants' contributions, participants occasionally described very in‐depth, emotive and detailed experiences, and extended time was required to ensure participants felt that these experiences were sufficiently appreciated by the group. There was a sense that the working groups provided a welcome opportunity for participants to describe in‐depth significant challenges or emotional experiences that had until this point been unheard of. For service users and carers, this related to negative care experiences, and for staff, this related to systematic issues or issues with a particular service. This process of extensive disclosure was well‐managed in the working groups but could contribute to a group losing focus and sight of the overall aim of the project. To mitigate this, the facilitator provided participants with the objectives for each session to reduce repetitive sharing of care experiences and move forward with the project aim.
*Facilitator: I appreciate the storytelling because it gives context to why we're doing the things we're doing. I'm also mindful that our time is short, we have today and we have two more sessions. We really do want to start working and we need to nail down the three themes.*
  (Group 1; Session 3)
  Similarly, within the individual interviews, some participants implied that some forms of sharing can be repetitive, reflect complaining, or consume a relatively large proportion of time:
*Carer (P7): Sometimes you might find it is the same 2 or 3 that are having an input.*
  Carer interview (Group 2; after Session 1)
3. **The scope of the group as an invisible barrier**
  This theme reflects the challenge of the scope of a smaller‐scale project. The agreed consensus between service users, staff, carers and the facilitator is that there are certain elements of a service that cannot be impacted within the scope of this group. Occasionally, the scope of the group resulted in participants questioning their service improvement and the impact that they could have:
*Staff (P4): How much control have we of changing the likes of discharge reports from the hospital? You know that, that's a huge*.
*Staff (P7): Yeah, I was thinking that.*
  (Group 2; Session 2)
*Facilitator: Also, you know, the delay of getting results back to the GP if you were seen in hospital, that's an issue as well. If we think about what we can achieve in this group, is that something you want to look at or do we feed back and say we raised it?*
*Staff (P5): We probably would need somebody from the GP service and the hospitals for it to be effective. It's probably something we feedback*.
*Service User (P2): The problem, the problem is that the computer, computers aren't compatible…*
*Facilitator: So even as it is the community and the hospital can't see each others, they're not integrated. So there is work being done, but I think it's bigger than this group as well*.
*Service user (P2): I think so.*
  (Group 1; Session 2)
  This style of discussion was most evident in the staff members' discourse but also observed among some service users. Additionally, the scope of the group also influenced how some participants ranked priorities during the prioritisation activity:
*Service User (P6): Prioritisation. Yeah, yeah. Yeah, it did. It was very. It was very visual in that we had a sheet with themes that we had come up with over past discussion. We put up the yellow stickers on those things that we could do something about there. There were other things that we had discussed but realised this group, there actually isn't anything we could do about those. They need to be fed back, yeah, to the Hub and management*.
  Service user interview (Group 1; after Session 2)
*Staff (P4): But in the back of my mind, I was like what can we change, that affected my choice*.
  (Group 1; Session 2)
  The scope of the group can be a barrier as it may have affected participants' confidence in their ability to develop and implement their service improvement. Additionally, it may have influenced some of the individuals' decision‐making when it came to choosing the issues they wished to address. Importantly, one staff member noted that the mix of service users and staff hindered their engagement in the initial stages:
*Staff (P7): There were some things that I wanted to say more of and I felt I couldn't…. I felt I couldn't really say that in front of the service users. If it had just been myself and erm staff maybe I could have said it, but I did feel I had to hold back a little bit…*
*maybe if I had been given the chance to erm to voice something maybe privately to [facilitator] you know. But then I wouldn't really… it's not what we are about, it's about coming together as a group*.
  Staff interview (Group 2; after Session 1)
  However, this was not a prominent theme across the full dataset and there was a sense that staff felt more comfortable in sharing as the participants' cooperative relationships were established.

## 4 Discussion

This study identified barriers and enablers to EBCD implementation, specifically in the context of ECC. Prior literature has largely focused on individual health conditions or condition‐specific healthcare settings; our study demonstrates that the EBCD process can be applied effectively in integrated care settings like Ireland's ECC programme, to develop health service improvements with consensus between diverse service users and health and social care staff.

Our findings align with Ramos et al.'s [10] report that the facilitator has a critical role to play in the success of the EBCD process as they coordinate the project and connect the organisation, staff and patients. Our analysis suggests that while there are advantages in having a healthcare professional as a facilitator, internal facilitators must continually reflect on their own positionality to maintain the facilitator role in a context where they could equally hold a participant role. This is perhaps especially important where working groups, including both service users and staff, together, rather than separate working groups, are conducted. Conversely, external facilitators could offer neutrality and objectivity, which can create safer spaces for dialogue and help balance power dynamics. Their expertise in group facilitation can enhance co‐design by guiding discussions and fostering collaboration [15]. Their lack of familiarity with the healthcare setting and specific participant contexts may limit their ability to develop practical recommendations, underlining the importance of facilitators' understanding of the local context [16]. External facilitators may face challenges in building trust [17], which may be especially challenging in accelerated projects where time to establish rapport is limited. The financial and logistical burden of external facilitation, given its variability in cost and intensity, may restrict its feasibility for some projects [15]. Ultimately, there are merits and limitations to both internal and external facilitation.

Further, the facilitator role may be especially crucial when different participants have competing priorities. In some instances, service users may recognise that their experiential knowledge has limited bearing on, for example, the evaluation of new therapies [18]; in other instances, owing to the power dynamics discussed earlier, service users may defer to staff participants even when their experiential knowledge is highly valued. The facilitator needs to ensure all participants have had the opportunity to contribute to priority‐setting, and where competing priorities are identified, can invite participants to consider *how* they will resolve this. The group might agree to finalise priorities based on a majority consensus, or by reflecting on feasibility, urgency of implementation or some other dimension, before revising the priorities. Facilitating discussion of how to resolve competing priorities will ensure group members feel listened to, even if their personal priority is not ultimately selected.

Prior studies [19] have identified staff disengagement as a barrier to the EBCD process; this was not the case in our study. This difference might be explained by the fact that the ECC scheme is a new service, offering opportunities for new ideas and collaboration among new teams of healthcare professionals. Possibly, it is easier to establish an EBCD project in a new setting, where staff may have an expectation of developing service improvements as these are rolled out. In contrast, more established services might be more likely to experience staff disengagement or indeed burn‐out as a challenge to service improvement projects. Other likely contributors to the high staff engagement in this project include the voluntary nature of participation and the relatively comparable career stages of the health and social care professionals involved, which potentially reduced issues of hierarchy noted elsewhere [20].

Another key finding was that participants were mindful of the system‐level constraints around the potential for change in healthcare experiences; at times, this constrained their own contribution as they wanted to prioritise making contributions that were likely to result in solutions. To combat this in our study, participants were reassured that their suggestions remained valuable, and would be relayed to management, even when a direct solution could not be generated at that time, an approach welcomed by the participants. Nonetheless, our analysis highlights a challenge for EBCD facilitators who want to encourage creative “blue skies” thinking among participants but who also want to ensure participants see the value and impact of their participation by realising pragmatic and feasible solutions. This theme reflects participant awareness of the systems‐level barriers identified in previous research [10]. Committing to valuing and relaying all suggestions, even in a resource‐constrained environment, could ensure that participants are forthcoming and creative in their suggested solutions. Further, while this was not a key theme in our data, the potential drawbacks of mixed staff and service user working groups were noted. Despite this, the groups were successful in developing service improvements, suggesting that even where members articulate reservations about joint working groups, unless these reservations are significant or widespread, the EBCD process will likely still be effective.

This study is enhanced by the analysis of two forms of qualitative data (the co‐design working group transcripts and individual brief interviews). The analysis was led by a team who were not involved in (but were familiar with) the EBCD process or in healthcare services delivery; this allowed us the distance needed to critically reflect on potential barriers and enablers to the EBCD process. One potential issue is that the co‐design working groups were highly collegial, and the power imbalance described in prior research [9, 10], though not invisible, was not especially prominent. Therefore, our analysis is not instructive in terms of managing this power imbalance or in managing potential conflicts among group members. Nonetheless, our findings generate recommendations for the implementation of ECBD approaches in ECC.

## 5 Conclusions and Recommendations

The EBCD approach resulted in actionable service improvements, which can be applied across services in Ireland, demonstrating that it is a useful approach in the ECC context. Several recommendations are generated from our findings. First, and unsurprisingly, EBCD projects require resources. Identifying the personnel, time and financial costs of facilitating workshops and evaluating outcomes is crucial before embarking on an EBCD project. Second, we recommend that the optimal facilitator(s) is an *experienced, informed, but removed*, facilitator, who has the service knowledge to engage with group members (e.g., through engagement in similar projects elsewhere), but who is not so closely affiliated with the service as to magnify power dynamics among mixed group members. Facilitators need to adopt a flexible approach as participants can have different preferences (e.g., in terms of modes of communication or engagement between working group meetings). We acknowledge that owing to resource constraints, the facilitator may well be internal. Although co‐facilitation with service users is often perceived as optimal and can partly address power dynamics, service users may not have the requisite facilitation experience and require support to develop facilitation skills. Thus, we recommend that facilitation skills be prioritised over ‘type’ of facilitator. Next, we recommend bringing staff and service users together even at the initial stage (which is sometimes done separately), as this fosters rapport from the outset and facilitates the sharing of experiences and perspectives. Finally, to evaluate EBCD implementation, we recommend facilitating access to both working group transcripts, and individual evaluations of EBCD engagement for evidence triangulation, where resources allow.

## Author Contributions

**Fay O'Donoghue:** writing – original draft, writing – review and editing, formal analysis, data curation, visualisation, validation, methodology. **Máire T O'Donnell:** conceptualisation, writing – review and editing, methodology, project administration. **Tomás P Griffin:** writing – review and editing, visualisation, supervision, resources. **Eileen Fahy:** investigation, data curation, project administration, writing – review and editing. **Elaine Newell:** conceptualisation, methodology, investigation, funding acquisition, writing – original draft, writing – review and editing, project administration, data curation, resources, supervision. **Ann‐Marie Creaven:** supervision, writing – original draft, writing – review and editing, methodology, formal analysis.

## Ethics Statement

This study received ethical approval from the Galway Clinical Research Ethics Committee (CA 2902).

## Consent

All service users gave informed consent to participate in the study.

## Conflicts of Interest

The authors declare no conflict of interest.

## Supporting information

Supporting information.

## Data Availability

The data from this study are not available. Owing to the small number of participants and the specific sites they were recruited from, there could be a risk of identification from the transcripts.
