# Supplementary material for: Enablers and Barriers to an Experience‐Based Co‐Design Process to Develop Service Improvements in Enhanced Community Care in Ireland: A Qualitative Study
Source: Health Expect. 2025 Mar 12;28(2):e70206. doi: 10.1111/hex.70206 (PMC11898217; doi:10.1111/hex.70206)
Supplement: Supplementary file 1 — Supporting information. [file HEX-28-e70206-s001.docx]

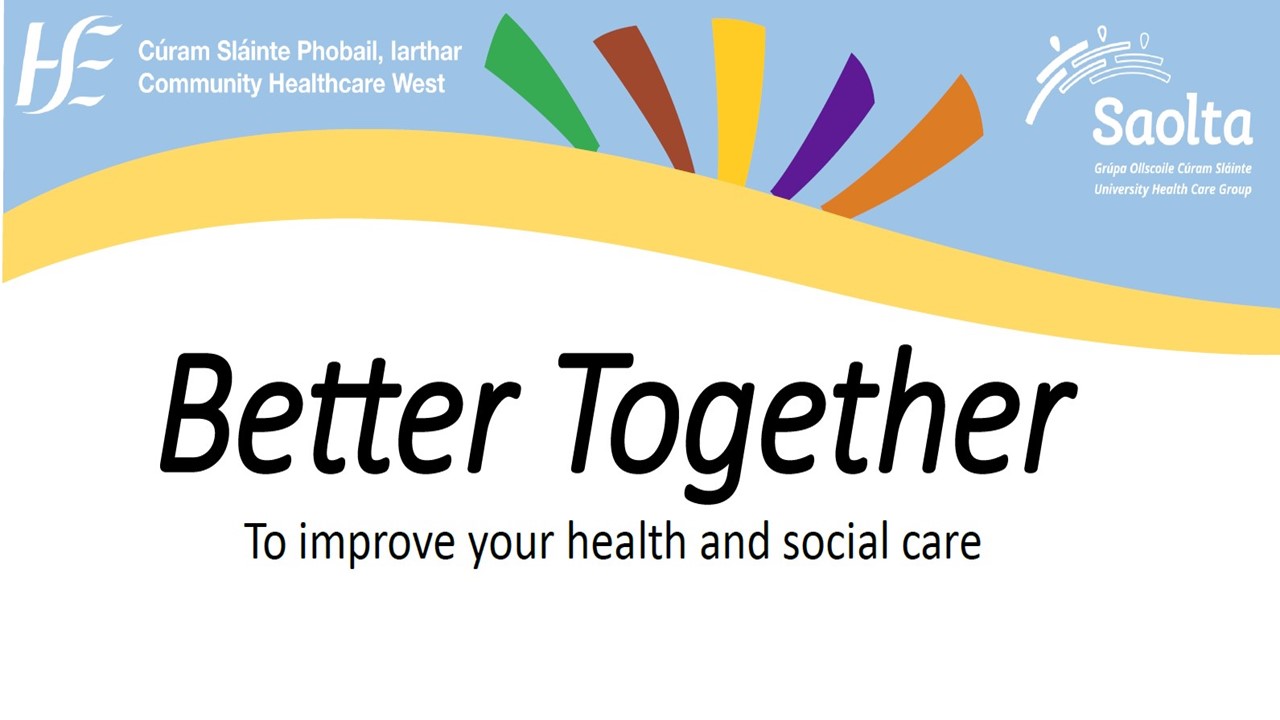


**Sharing your story- key points about your care experience**

This resource can help you organise your thoughts. Only share what you want to share.

1. What went well during your care experience?

________________________________________________________________________________________________________________________________________________________________________________________________________________________________________________________________________________________________________________________________________________________________________________________________

1. What did not go well with your care experience?

________________________________________________________________________________________________________________________________________________________________________________________________________________________________________________________________________________________________________________________________________________________________________________________________

1. Do you have any ideas/suggestions could have improved your care experience?

________________________________________________________________

________________________________________________________________

________________________________________________________________

________________________________________________________________

________________________________________________________________

________________________________________________________________
